# Successful sequential management of traumatic choledochal leak and stenosis in children using ERCP: a case report and literature review

**DOI:** 10.3389/fmed.2024.1446371

**Published:** 2024-11-06

**Authors:** Cuo Leng, Yu Zou, Zhoujian Yang, Xinhua Zhao

**Affiliations:** ^1^Department of Clinical Medicine, North Sichuan Medical College, Nanchong, China; ^2^Department of Gastroenterology, Mianyang Central Hospital, Mianyang, China; ^3^Department of Pediatrics, Mianyang Central Hospital, Mianyang, China

**Keywords:** ERCP, child, trauma, choledochal leak, choledochal stenosis

## Abstract

Traumatic Choledochal Leak and Stenosis in Children is a relatively rare bile leakage, and there is no report of endoscopic retrograde cholangiopancreatography [ERCP] bile duct stent treatment in 3 years old, which avoids procedure and biliary tract modifications after the failure of conservative treatment of bile leakage, and solves the previous need for procedure in a minimally invasive or even non-invasive way. At the same time, it can be concluded that identifying the signs of conservative treatment failure is important, and ERCP is superior to CT and MR in diagnosing traumatic biliary leak in young children. Traumatic bile leakage common bile duct inflammatory stenosis, brittle tissue, not suitable for expansion, the choice of 5 Fr to 10 Fr plastic stent sequential treatment is an effective regimen. At the same time, it is necessary to closely monitor the biliary patency after ERCP to understand the long-term postoperative efficacy. Comprehensive evaluation before ERCP and detailed post ERCP monitoring require the participation of pediatricians.

## Introduction

Biliary fistula is the leakage of bile or bile-containing fluid from a break in the biliary system into the abdominal cavity or outside the body (1). Common causes of biliary fistula include hepatobiliary procedure, trauma, inflammation, and neoplasms (2). The treatment of biliary fistula encompasses conservative medical treatment, interventional treatment and traditional surgical treatment. However, surgical treatment is highly invasive and can have a mortality rate of up to 6.67% (3), while ERCP is superior to procedure in terms of the reoperation rate, complication rate, and length of hospital stay (4). Recent reports state that three children aged 10 to 15 years were treated with early ERCP stenting, which found higher-grade liver lacerations were associated with higher bile leak complications (5). In the past, most cases of traumatic bile leakage in children were cured after stent placement, or were treated by surgery with drainage due to the severity of recurrent disease, so lack of experience with ERCP again after relapse. For managing a bile leak in such a young patient ERCP is superior to CT and MR in diagnosing traumatic biliary leak in young children. Traumatic bile leakage common bile duct inflammatory stenosis, brittle tissue, not suitable for expansion, the choice of 5 Fr to 10 Fr plastic stent sequential treatment is an effective regimen. At the same time, it is necessary to closely monitor the biliary patency after ERCP to understand the long-term postoperative efficacy.

## Case report

A three-year-old boy was presented to our hospital with liver rupture 5 h after falling from a tricycle. He exhibited drowsiness, abdominal distention, swelling in the left upper limb, palpable bone friction in the left clavicle area, and out-of-hospital abdominal ultrasound and enhanced computed tomography (CT) revealed a rupture of the right posterior lobe of the liver, posterior hematoma, spleen rupture, multiple fractures, and fluid with mixed densities in the peritoneal cavity. The patient received conservative treatment in the intensive care unit, with two peritoneal drainage catheters inserted to drain reddish-brown fluid due to progressive abdominal distension, white clay-colored stool, and darkening urine. After a period of 19 days, ascites resolved, and abdominal distension did not worsen; consequently, the family requested discharge against medical advice.

**Figure 1:**
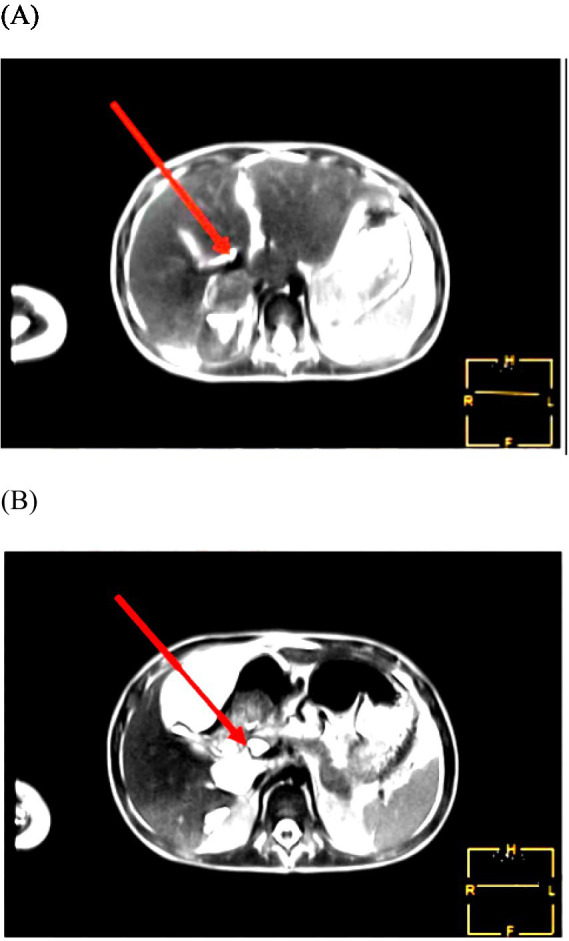
(A,B) MRCP indicated mild dilatation of the extrahepatic and intrahepatic bile ducts (A), with a nodular watery signal shadow observed at the head of the pancreatic segment of the common bile duct, suggestive of a cyst or bile retention (B).

**Figure 2:**
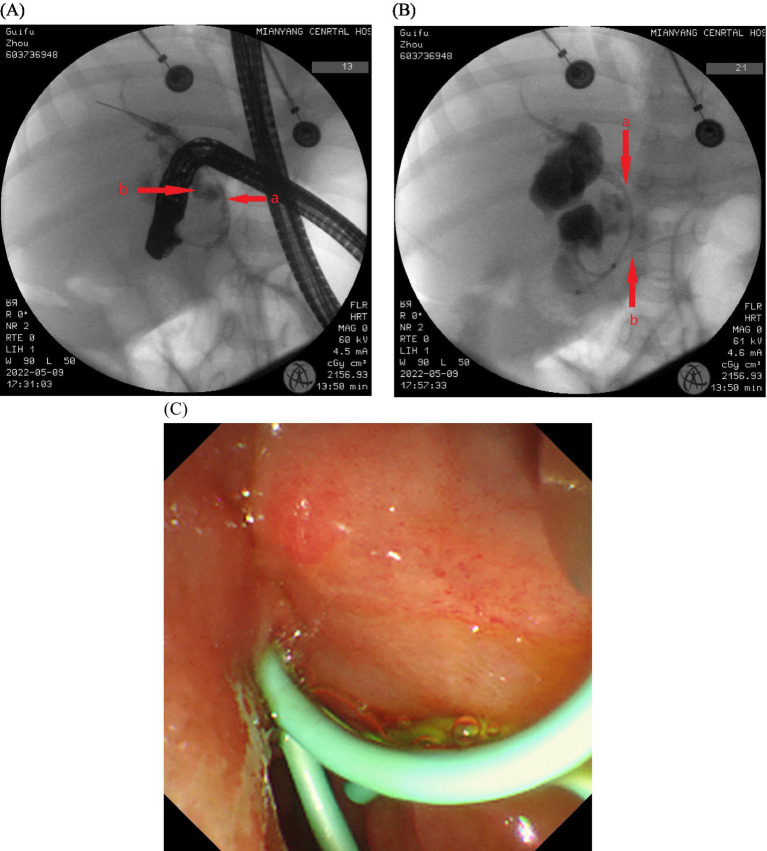
(A–C) The ERCP intubation was difficult, the bile duct was successfully inserted by double guide wire, Arch knife cholangiography revealed obvious narrowing of the middle and lower sections of the common bile duct (Aa), With visible contrast extravasation outside the bile duct, and local contrast agent accumulation (Ab). Neither the contrast catheter nor the arch incision knife could pass through the bile duct stenosis. The 5 Fr biliary-pancreatic duct stent was tried to pass through the bile duct stenosis, and the 5 Fr biliary-pancreatic duct stent (5 cm, 5 Fr, single pig tail stent) was placed in the bile duct to drainage bile (Ba), and the pancreatic duct stent was placed in the main pancreatic duct to prevent postoperative pancreatitis (Bb). Smooth bile drainage was achieved (C).

After 2 days of discharge, the patient presented with an increased abdominal circumference. Abdominal ultrasound revealed a large amount of ascites effusion, and the intraperitoneal catheter drained yellow-brown fluid. Following 5 days of conservative treatment, the effusion improved, and no further fluid drainage was required. Subsequent magnetic resonance cholangiopancreatography [MRCP] indicated mild dilatation of the extrahepatic and intrahepatic bile ducts (Figure 1A), with a nodular watery signal shadow observed at the head of the pancreatic segment of the common bile duct, suggestive of a cyst or bile retention (Figure 1B). Compared with the initial admission, abdominal enhanced CT revealed slight enlargement, with minimal to moderate effusion in the upper abdominal cavity. Ultrasound-guided abdominal puncture and drainage of pale yellow ascites were performed, accompanied by a recurrence of white clay-colored stool, raising concerns for a possible choledochal leak. Fifteen days after admission, the patient continued to experience abdominal distension with poor drainage efficacy. On the 38th day after the injury, ERCP with bile duct and pancreatic duct stenting was performed. However, the ERCP intubation was difficult, the bile duct was successfully inserted by double guide wire, Arch knife cholangiography revealed obvious narrowing of the middle and lower sections of the common bile duct (Figure 2Aa), With visible contrast extravasation outside the bile duct, and local contrast agent accumulation (Figure 2Ab). Neither the contrast catheter nor the arch incision knife could pass through the bile duct stenosis. The 5 Fr biliary-pancreatic duct stent was tried to pass through the bile duct stenosis, and the 5 Fr biliary-pancreatic duct stent (5 cm, 5 Fr, single pig tail stent) was placed in the bile duct to drainage bile (Figure 2Ba), and the pancreatic duct stent was placed in the main pancreatic duct to prevent postoperative pancreatitis (Figure 2Bb). Smooth bile drainage was achieved (Figure 2C). Postoperative diagnosis confirmed middle and lower common bile duct stenosis with biliary fistula. No fluid drainage was required 2 days after the operation, and abdominal pain subsided after 4 days, accompanied by the presence of yellow bowel movements and absence of complications.

**Figure 3:**
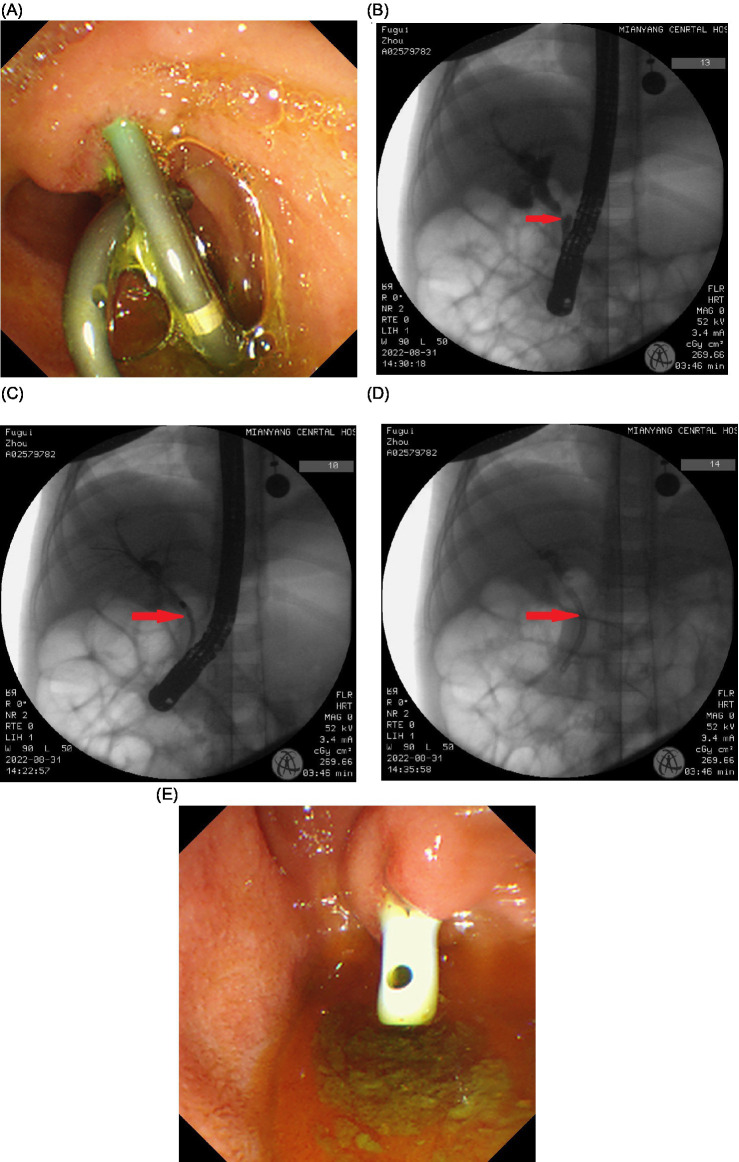
(A–E) Three months after the first ERCP, the second ERCP was performed and the bile duct stent and pancreatic duct stent were in place and patent (A). After removing the stent, the bile duct was successfully inserted, cholangiography revealed an annular stenosis in the middle and lower parts of the common bile duct, approximately 2.5 mm in length (B). The bow knife successfully traversed the stricture segment, and there was no significant dilation of the intrahepatic and extrahepatic bile ducts. Dilators (7.5 Fr and 10 Fr) were utilized to expand the bile duct stricture segment (C). Subsequently, a plastic bile duct stent (10 Fr, 5 cm) was placed in the common bile duct (D) to ensure unobstructed bile drainage (E).

**Figure 4:**
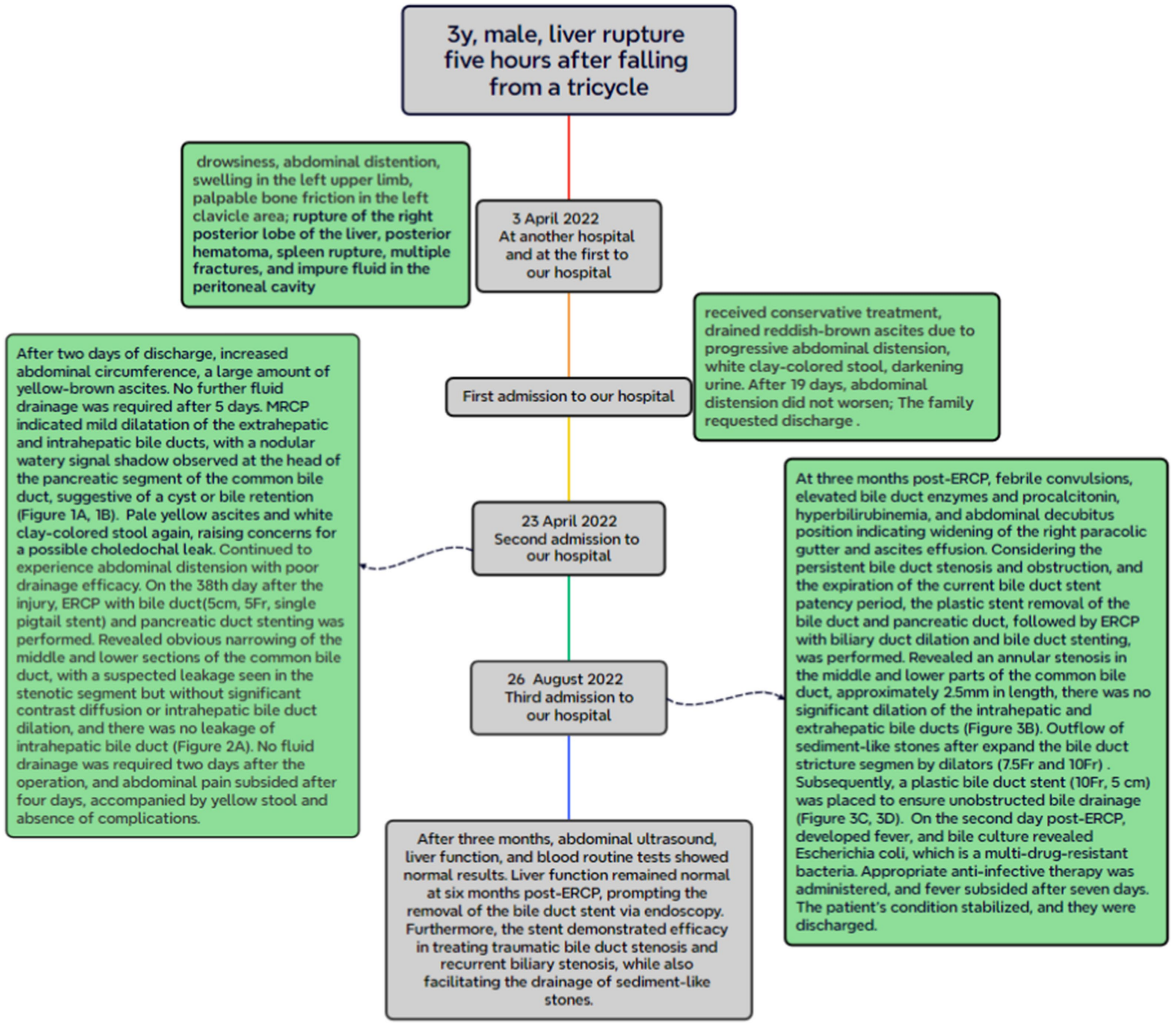
Timeline of this case.

At 3 months post-ERCP, the patient presented with febrile convulsions, elevated bile duct enzymes, elevated procalcitonin, hyperbilirubinemia, and abdominal decubitus position (including kidney–ureter-bladder imaging) indicating widening of the right paracolic gutter and ascites effusion. Considering the persistent bile duct stenosis and obstruction, and the expiration of the current bile duct stent patency period, the plastic stent removal of the bile duct and pancreatic duct, followed by ERCP with biliary duct dilation and bile duct stenting, was performed. The bile duct stent and pancreatic duct stent were in place and patent (Figure 3A). After removing the stent, the bile duct was successfully inserted, cholangiography revealed an annular stenosis in the middle and lower parts of the common bile duct, approximately 2.5 mm in length (Figure 3B). The bow knife successfully traversed the stricture segment, and there was no significant dilation of the intrahepatic and extrahepatic bile ducts. Dilators (7.5 Fr and 10 Fr) were utilized to expand the bile duct stricture segment (Figure 3C). Subsequently, a plastic bile duct stent (10 Fr, 5 cm) was placed in the common bile duct (Figure 3D) to ensure unobstructed bile drainage (Figure 3E). Postoperative diagnosis included middle and lower bile duct stenosis, with the implantation of bile duct and pancreatic duct stents. On the second day post-operation, the patient developed fever, and bile culture revealed *Escherichia coli*, which is a multi-drug-resistant bacteria. Appropriate anti-infective therapy was administered, and fever subsided after 7 days. The patient’s condition stabilized, and the patient (he) was discharged (Figure 4).

After 3 months, abdominal ultrasound, liver function, and blood routine tests showed normal results. Liver function remained normal at 6 months post-operation, prompting the removal of the bile duct stent via endoscopy. The procedure yielded positive outcomes, effectively curing the bile leakage. Furthermore, the stent demonstrated efficacy in treating traumatic bile duct stenosis and recurrent biliary stenosis, while also facilitating the drainage of sediment-like stones (Figure 4).

## Discussion

We present a case describing the successful sequential treatment of traumatic common bile duct leakage with common bile duct stenosis in a 3-year-old child. For managing a bile leak in such a young patient. Identifying the signs of conservative treatment failure is important. ERCP is superior to CT and MR in diagnosing traumatic biliary leak in young children. Traumatic bile leakage common bile duct inflammatory stenosis, brittle tissue, not suitable for expansion, the choice of 5 Fr to 10 Fr plastic stent sequential treatment is an effective regimen. In the past, most cases of traumatic bile leakage in children were cured after stent placement, or were treated by surgery with drainage due to the severity of recurrent disease, so lack of experience with ERCP again after relapse. The patient developed febrile seizures and abnormal liver function after three initial ERCP treatments, review of the cases found that the patient was not monitored for about 3 months after ERCP, therefore, it is recommended to monitor the biliary patency after ERCP according to the condition and the patient’s wishes to understand the long-term postoperative efficacy.

Meanwhile, clinical practice often involves various unpredictable combinations of bile leakage and benign bile duct stricture, along with diverse complications. To delve further into this, we conducted a literature review aimed at discussing the current efficacy of ERCP bile duct stents in treating bile leakage, managing comorbidities in patients with traumatic biliary fistula, and assessing prognosis. Wang et al. reported on 42 patients with bile leakage following traumatic liver rupture, among whom 10 patients underwent surgical treatment, while 32 patients underwent biliary stent drainage. The reoperation rate was 30.0% in the surgical group, with an average postoperative hospital stay of (32.6 ± 18.6) days, whereas the reoperation rate was 6.3% in the stent group, with an average length of hospital stay of (20.2 ± 8.3) days. Complications occurred in 50% of the surgical group and 15.6% of the stent group, respectively. Complications in the stent group included hyperamylasemia, acute pancreatitis, and duodenal papillae hemorrhage, all of which were successfully treated with symptomatic supportive measures such as enzyme suppression, anti-infection, fluid rehydration, and hemostasis. Follow-up of all cases for 1 to 2 years revealed no occurrences of cholangitis or biliary stricture, and no instances of stent displacement or blockage were found (4). Zhong et al. reported on the safety and efficacy of ERCP combined with intraperitoneal catheter drainage in the treatment of 24 patients with bile leakage after hepatobiliary procedure. The treatment demonstrated a high cure rate, particularly for stump of cystic duct leakage. Depending on the size of the contrast leak or the quality of abdominal drainage, endoscopic nasobiliary drainage [ENBD] can be selected when the leakage is large (abdominal drainage volume > 300 ml), whereas endoscopic retrograde biliary drainage [ERBD] is chosen if the leakage is small. ENBD combined with ERBD can be employed to increase the cure rate of bile leakage when necessary (6). Wei et al. reported on 9 cases of cystic duct leakage and 10 cases of intrahepatic bile leakage after procedure. All cases were treated with sphincterotomy combined with bile duct stenting. Endoscopic cholangiography was repeated 12 weeks later. One case in the cystic duct leakage group required endoscopic intervention again, while several replacements were necessary in the intrahepatic bile leakage group. Following multiple endoscopic treatments, no abnormal manifestations were observed during the 1-year follow-up. In summary, ERCP bile duct stents are effective in treating bile leakage, with the location of the bile leakage possibly influencing the success of the initial ERCP (7). Mounsey et al. reported the discovery of a traumatic biliary fistula on day 7 of hospitalization in a 37-year-old woman who had undergone Roux-en-Y gastric bypass procedure. They recommended repeat imaging of high-grade liver injury between days 7 and 10 to evaluate for delayed bile leakage or other complications. For patients with Roux-en-Y anatomy, a newer approach involves EUS-directed transgastric ERCP [EDGE] and deployment of a luminal butt metal stent to establish a fistula between the gastric pouch and the residual stomach to complete ERCP (8).

About the effectiveness of ERCP bile duct stents in treating post-traumatic bile duct strictures, as well as the prevention and management of complications such as restenosis after benign bile duct strictures and bile duct stones, are also discussed. This case has undergone ERCP for half a year, with positive postoperative outcomes. Kaka Ali et al. reported on a patient presenting with epigastric pain, anorexia, and scleral jaundice following laparoscopic cholecystectomy. Suspected cholangiocarcinoma was diagnosed after magnetic resonance cholangiopancreatography, with postoperative pathology revealing bile duct traumatic neuroma. Therefore, patients with a history of biliary trauma can undergo dynamic liver magnetic resonance imaging [MRI], cholangioscopy, and endoscopic ultrasound biopsy to identify the cause of post-traumatic bile duct stricture and avoid unnecessary procedure (9). Costamagna et al. conducted a study involving 42 patients with postoperative biliary stricture who were followed up for 10 years with multiple endoscopic stenting. Four cases experienced biliary stricture reconstitution after an average of 6.8 years, and three cases required extraction of common bile duct stones. After a mean follow-up of 7.1 years (ranging from 2.5 to 12.1 years), no strictures or recurrence of bile duct stones were observed. Hence, patients with biliary tract stricture and cholelithiasis after ERCP treatment for biliary tract injury can undergo multiple ERCP procedures. It’s noteworthy that causes of recurrent stricture include incomplete treatment of the stricture site shown on X-rays and failure to diagnose right posterolateral bile duct stenosis during initial treatment. The rate of recurrent stenosis can be minimized by reducing missed diagnoses, and re-ERCP is effective for unavoidable recurrent strictures (10). Thai Binh et al. reported that fibrosis can lead to the recurrence of bile duct strictures. They found that percutaneous transhepatic endoscopic thulium laser vaporesection has a 100 percent immediate and short-term technical success rate for managing severe and focal benign biliary strictures (11). Ishikawa et al. described two cases of refractory benign biliary stricture [BBS] treated with endoscopic ultrasound-guided choledochoduodenostomy [EUS-CDS] fistulas. After stent removal, the patients no longer required them, and these fistulas remained patent for more than 2.5 years without cholangitis. The creation of fistulas using EUS-CDS is thus considered an effective treatment option for BBS (12). Jang et al. reported a high success rate in treating BBS with fully coated self-expanding metal bracket [FCSEMS], although recurrence is possible. They also suggested that corticosteroids may be useful due to their antifibrotic and anti-inflammatory effects. In a porcine BBS model, steroid-eluting FCSEMS has shown potential as a safe and effective treatment modality to reduce fibrotic tissue (13). Maatman et al. reported that a quarter of 743 patients with necrotizing pancreatitis developed biliary strictures after more than 1 year. They found that 20 percent of patients with biliary strictures required surgical correction. Predictors of unsuccessful endoscopic therapy included a duration of necrotizing pancreatitis lasting more than 6 months or a history of infectious necrosis (14). Li et al. documented the cases of 9 patients with BBS unresponsive to conventional treatments. These individuals underwent percutaneous transhepatic biliary drainage [PTBD] and ERCP prior to undergoing magnetic compression anastomosis [MCA]. The MCA procedure involved delivering the sub-magnet of the MCA device to the proximal end of the obstruction via the PTBD pathway, while the parent-magnet was delivered to the distal end of the obstruction via ERCP. After recanalization, the magnets were withdrawn, and the stent was removed following at least 6 months of biliary stenting (or PTBD). Follow-up examinations conducted for 2 to 66 months revealed no occurrence of stenosis without stenting. MCA has proven effective in treating severe BBS cases that do not respond to conventional methods (15).

Advanced techniques like EDGE for anatomical variations, thulium laser vaporization, steroid-eluting FCSEMS for reducing biliary fibrosis, and MCA for severe BBS exemplify technological progress. Therefore, further research is crucial to refine and expand endoscopic treatment options for bile leakage and biliary stricture. Strategies to reduce stricture recurrence include improving preoperative diagnostic accuracy, optimizing intervention strategies, and identifying predictors of unsuccessful endoscopic therapy.

About pediatric cases, Ali et al. reported an 11-year-old boy with grade 4 liver injury extending onto the porta-hepatis along with gross ascites, sepsis, who underwent subhepatic and pelvic peritoneal drain placement which showed frank bile and pus. Although his sepsis improved with antibiotics, his subhepatic drain outputs remained high. The child underwent ERCP with ampullary sphincterotomy and biliary stent placement, the post-ERCP effect was obvious, and the bile leakage was cured (16). Pulliam et al. reported a 13-year-old male after traumatic ERCP demonstrating a narrow, but otherwise normal, extrahepatic bile duct with findings of a deep right lobe intrahepatic laceration and bile leak with contrast extravasation tracking to the dome of the liver. Plastic biliary stents of different sizes were placed in the extrahepatic biliary tree to drain the bile, the tip of the stent extended from 2 to 3 cm downstream of the biliary defect, and the bile leakage persisted. After 10 days, a large amount of bile was still drained. Therefore, a joint plan was developed to reattempt ERCP to gain direct access to the bile leak for intraductal glue embolization – a joint procedure between gastroenterology and interventional radiology, and a short biliary stent was placed to solve the possible, unvisualized secondary site of bile leakage, so that the bile leakage was cured (17).

## Conclusion

For managing a bile leak in such a young patient. Identifying the signs of conservative treatment failure is important. ERCP is superior to CT and MR in diagnosing traumatic biliary leak in young children. Traumatic bile leakage common bile duct inflammatory stenosis, brittle tissue, not suitable for expansion, the choice of 5 Fr to 10 Fr plastic stent sequential treatment is an effective regimen. At the same time, it is necessary to closely monitor the biliary patency after ERCP to understand the long-term postoperative efficacy.

## Patient perspective

The patient’s family expressed great satisfaction with the ERCP diagnosis and stent sequential treatment of biliary leakage with biliary stricture.

## Data Availability

The original contributions presented in the study are included in the article/supplementary material, further inquiries can be directed to the corresponding author.
